# Cellular signaling mediated by nitrated cyclic nucleotide and regulated by hydrogen sulfide

**DOI:** 10.1186/1471-2210-11-S1-O23

**Published:** 2011-08-01

**Authors:** Takaaki Akaike

**Affiliations:** 1 Department of Microbiology, Graduate School of Medical Sciences, Kumamoto University, Japan

## Background

We have discovered earlier 8-nitroguanosine 3**’**,5’-cyclic monophosphate (8-nitro-cGMP) formed as a second messenger for nitric oxide (NO) and reactive oxygen species (ROS), mediating a unique electrophilic cellular signaling. 8-Nitro-cGMP is formed via NO and causes protein S-guanylation. The regulation mechanism for intracellular 8-nitro-cGMP formation and its metabolic fate still remain unclear, however.

## Results

Here we identified that hydrogen sulfide anion (SH-) rather than H2S gas per se is a potent negative regulator for endogenous 8-nitro-cGMP. SH- or free thiolate anion, effectively reacts with various 8-nitro-cGMP in cells to primarily generate each SH substituted, or sulfhydrated derivative via their direct chemical sulfhydration with SH-. This electrophile sulfhydration can therefore produce a new nucleophilic derivative, i.e., electrophile to nucleophile bioconversion to be caused by hydrogen sulfide. We also clarified that sulfhydration is functionally active in regulation of electrophilic cellular signaling, mediated by H-Ras oncogene. One of the major pharmacological effects of SH- was brought about by suppression of cellular senescence, caused by 8-nitro-cGMP-dependent Ras activation, involving downstream signaling molecules including Raf/Mek/Erk pathway finally activating p53 in cells.

## Conclusion

This may thus indicate an entirely novel aspect of biological functions of hydrogen sulfide in that it eventually behaves as an anion form, rather than a gaseous molecule, and interacts 8-nitro-cGMP to modulate its signaling functions. Moreover, we now consider that 8-nitro-cGMP is a major second messenger regulating electrophilic cellular signaling occurring widely in different species of biota including plants. The discovery of 8-nitro-cGMP and its signal modulator (i.e. hydrogen sulfide) may thus not only open a new era of research on the nucleotide cell signaling but also promote better understanding of the NO-mediated signaling mechanism in both eukaryotic and prokaryotic cells in general.

